# *De novo* genome assembly of the land snail *Candidula unifasciata* (Mollusca: Gastropoda)

**DOI:** 10.1093/g3journal/jkab180

**Published:** 2021-05-28

**Authors:** Luis J Chueca, Tilman Schell, Markus Pfenninger

**Affiliations:** 1 LOEWE-Centre for Translational Biodiversity Genomics (LOEWE-TBG), Senckenberg Nature Research Society, Frankfurt am Main 60325, Germany; 2 Department of Zoology and Animal Cell Biology, University of the Basque Country (UPV-EHU), Vitoria-Gasteiz 01006, Spain; 3 Senckenberg Biodiversity and Climate Research Centre (SBiK-F), Frankfurt am Main 60325, Germany; 4 Faculty of Biology, Institute of Organismic and Molecular Evolution (iOME), Johannes Gutenberg University, Mainz 55128, Germany

**Keywords:** annotation, *de novo* assembly, Geomitridae, land snails, long reads, molluscs, repeats

## Abstract

Among all molluscs, land snails are a scientifically and economically interesting group comprising edible species, alien species and agricultural pests. Yet, despite their high diversity, the number of genome drafts publicly available is still scarce. Here, we present the draft genome assembly of the land snail *Candidula unifasciata*, a widely distributed species along central Europe, belonging to the Geomitridae family, a highly diversified taxon in the Western-Palearctic region. We performed whole genome sequencing, assembly and annotation of an adult specimen based on PacBio and Oxford Nanopore long read sequences as well as Illumina data. A genome draft of about 1.29 Gb was generated with a N50 length of 246 kb. More than 60% of the assembled genome was identified as repetitive elements. In total, 22,464 protein-coding genes were identified in the genome, of which 62.27% were functionally annotated. This is the first assembled and annotated genome for a geometrid snail and will serve as reference for further evolutionary, genomic and population genetic studies of this important and interesting group.

## Introduction

Gastropods are the largest group among molluscs, representing almost 80% of the species in this second largest phylum. Within gastropods, snails occupy arguably the widest range of habitats of all metazoan taxa, ranging from deep sea vents to the alpine region and from deserts to polar regions. Land snail diversity is estimated around to 35,000 species (Solem 1984). Due to their low dispersal abilities, land snails have been employed long since in many evolutionary and population genetics studies (Cain and Sheppard 1954; Cameron 1992; Pfenninger *et al.* 1996; Chueca *et al.* 2017; Haponski *et al.* 2017). While these studies are mainly based on few loci, transcriptomes or mitochondrial genomes (Kang *et al.* 2016; Razkin *et al.* 2016; Romero *et al.* 2016; Korábek *et al.* 2019), only three whole nuclear genomes of land snails species are available so far, *Achatina fulica* (Guo *et al.* 2019), *Achatina immaculata* (Liu *et al.* 2021) and *Cepaea nemoralis* (Saenko *et al.* 2021).

**Figure 1:**
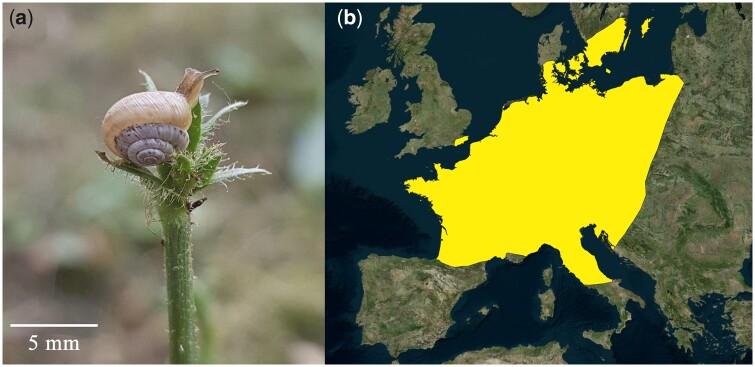
(A) Picture of an adult specimen of *C. unifasciata*, copyright © Luis J. Chueca. (B) Estimated extent of occurrence of *C. unifasciata* in Europe.

The Geomitridae are one of the most diverse families of molluscs in the Western-Palearctic region. The family is composed of small to medium-sized species, characterized by presenting several (reproductive) adaptations to xeric habitats (Giusti and Manganelli 1987). *Candidula unifasciata* (NCBI: txid100452) is a small land snail species (5–10 mm) widely distributed along western Europe, from southern France and Italy to central and northern Europe (Figure 1). *C. unifasciata* inhabits dry meadows and open lowlands with rocks, being also present in gardens and vineyards. A recent molecular revision of *Candidula* (Chueca *et al.* 2018) revealed the polyphyly of the genus, and split the species that composed it into six genera, questioning the traditional anatomical classification. Previous work focused on the speciation (Pfenninger and Magnin 2001; Pfenninger *et al.* 2003) and climate-driven evolution (Pfenninger and Posada 2002; Pfenninger *et al.* 2007) of the genus, however, in depth analyses were hampered by the lack of genomic markers. Although, there are many taxonomical, phylogeographical, and evolutionary studies concerning Geomitridae species (Pfenninger and Magnin 2001; Sauer and Hausdorf 2010; Brozzo *et al.* 2020), the lack of reference genomes makes it difficult to investigate deeper biological and evolutionary questions about geomitrids and other land snails species. Here, we present the annotated draft genome of *C. unifasciata* that will be a valuable resource for future genomic research of this important taxonomic group (Chueca *et al.* 2021).

## Materials and methods

### Sample collection, library construction, and sequencing

Several living individuals of *C. unifasciata* were collected from Winterscheid, Gilserberg, Gemany (50.93° N, 9.04° E). Genomic DNA was extracted independently from two specimens using the phenol/chloroform method and quality was checked by gel electrophoresis and NanoDrop ND-1000 spectrophotometer (LabTech, USA). Genomic DNA from the first specimen (size distribution = 20 kb) was employed for Illumina and PacBio sequencing. A total of 5.6 µg of DNA was sent to Novogene (UK) for library preparation and sequencing. Then, a 300 base pair (bp) insert DNA libraries was generated using NEBNext^®^ DNA Library Prep Kit and sequenced on 3 lanes of Illumina NovaSeq 6000 platform (150 bp paired-end [PE] reads). Quality of raw Illumina sequences was checked with FastQC (Andrews 2010). Low quality bases and adapter sequences were subsequently trimmed by Trimmomatic v0.39 (Bolger *et al.* 2014). For PacBio sequencing, a DNA library was prepared from 5 µg of DNA using the SMRTbell template prep kit v.1.0. Sequencing was carried out on 10 single-molecule real-time sequencing (SMRT) cells on an RSI instrument using P6-C4 chemistry.

To obtain Oxford Nanopore Technologies (ONT) long reads, we ran two flow cells on a MinION portable sequencer. Total genomic DNA from a second specimen (size distribution = 30 kb) was used for library preparation with the Ligation Sequencing kit (SQK-LSK109) from ONT, using the manufacturer’s protocols. Base calling of the reads from the two MinION flow cells was performed with guppy v4.0.11 (https://github.com/nanoporetech/pyguppyclient, last accessed on 07-06-2021), under default settings. Afterwards, ONT reads quality was checked with Nanoplot v1.28.1 (https://github.com/wdecoster/NanoPlot, last accessed on 07-06-2021) and reads shorter than 1000 bases and mean quality below seven were discarded by running Nanofilt v2.6.0 (https://github.com/wdecoster/nanofilt, last accessed on 07-06-2021).

Two living specimens, one adult and one juvenile, were kept in captivity for 3 days to purge the gut. After being purged, the whole bodies of both specimens were homogenized together into small pieces using steel balls and a Retsch Mill. Then, RNA was extracted following an standard Trizol extraction. The integrity of total RNA extracted was assessed on an Agilent 4200 TapeStation (Agilent, USA), after which, approximately 1 µg of the total RNA was processed using the Universal Plus mRNA-seq library preparation kit (NuGEN, Redwood City, CA, USA). Finally, the 300-bp insert size library was sequenced on a Illumina NovaSeq 6000 platform.

### Genome size estimation

Genome size was estimated following a flow cytometry protocol with propidium iodide-stained nuclei described in (Hare and Johnston 2012). Foot tissue of one fresh adult sample of *C. unifasciata* and neural tissue of the internal reference standard *Acheta domesticus* (female, 1 C = 2 Gb) was mixed and chopped with a razor blade in a petri dish containing 2 ml of ice-cold Galbraith buffer. The suspension was filtered through a 42-μm nylon mesh and stained with the intercalating fluorochrome propidium iodide (PI, Thermo Fisher Scientific) and treated with RNase II A (Sigma-Aldrich), each with a final concentration of 25 μg/ml. The mean red PI fluorescence signal of stained nuclei was quantified using a Beckman-Coulter CytoFLEX flow cytometer with a solid-state laser emitting at 488 nm. Fluorescence intensities of 5000 nuclei per sample were recorded. We used the software CytExpert 2.3 for histogram analyses. The total quantity of DNA in the sample was calculated as the ratio of the mean red fluorescence signal of the 2 C peak of the stained nuclei of the *C. unifasciata* sample divided by the mean fluorescence signal of the 2 C peak of the reference standard times the 1 C amount of DNA in the standard reference. Four replicates were measured to minimize possible random instrumental errors. Furthermore, we estimated the genome size by coverage from mapping reads used for genome assembly back to the assembly itself using backmap v0.3 (https://github.com/schellt/backmap; Schell *et al.* 2017, last accessed on 07-06-2021). In brief, the method divides the number of mapped nucleotides by the mode of the coverage distribution. By doing so, the length of collapsed regions with many fold increased coverage is taken into account.

### Genome assembly workflow

Several *de novo* genome assemblies were tested under different methods (see Supplementary Table S1). The pipeline, which showed the best genome, was selected to continue further analyses. The draft genome was constructed from PacBio long reads using wtdbg2 v2.5 (Ruan and Li 2020), followed by three polishing rounds of Racon 1.4.3 (Vaser *et al.* 2017) and three polishing rounds of Pilon 1.23 (Walker *et al.* 2014). After that, Illumina and PacBio reads were aligned to the assembly using backmap.pl v0.3 to evaluate coverage distribution. Then, Purge Haplotigs (Roach *et al.* 2018) was employed, under default parameters and cutoff values of 15, 72, and 160 to identify and remove redundant contigs.

### Scaffolding and gap closing

To further improve the assembly, we applied two rounds of scaffolding and gap closing to the selected genome assembly. The genome was first scaffolded with the PacBio and ONT reads by LINKS v1.8.7 (Warren *et al.* 2015) and then with RNA reads by Rascaf v1.0.2 (Song *et al.* 2016). After each scaffolding step Long-Read Gapcloser v1.0 (Xu *et al.* 2019) was run once, followed by three rounds of polishing former gap regions using Racon v1.4.3. BlobTools v.1.0 (Kumar *et al.* 2013; Laetsch and Blaxter 2017) was employed to screen genome assembly for potential contamination by evaluating coverage, GC content and sequence similarity against the NCBI nt database of each sequence. The resulting assembly was compared in terms of contiguity using Quast v5.0.2 (Gurevich *et al.* 2013), and evaluated for completeness by BUSCO v3.0.2 (Simão *et al.* 2015) against metazoa_odb9 data set.

### Transcriptome assembly

RNA reads were checked for quality and trimmed, as was explained above. The transcriptome was assembled using Trinity v2.9.1 (Haas *et al.* 2013). Then, the transcriptome assembly was evaluated for completeness by BUSCO v3.0.2 against the metazoa_odb9 data set. Moreover, the clean RNA-seq reads from different specimens were aligned against the draft assembly by HISAT2 v2.1.0 (Kim *et al.* 2015).

### Repeat annotation

RepeatModeler v2.0 (Smit and Hubley 2008) was run to construct a *de novo* repeat library from the genome assembly. The resulting repeat library was employed by RepeatMasker v4.1.0 (http://www.repeatmasker.org/, last accessed on 07-06-2021) to annotate and mask the genome assembly.

### Gene prediction and functional annotation

Genes were predicted by using different methods. First, genes models were predicted *ab initio* based on SNAP v. 2006-07-28 (Korf 2004) and the candidate coding regions within the assembled transcript were identified with TransDecoder v5.5.0 (https://github.com/TransDecoder/, last accessed on 07-06-2021). Second, we used homology-based gene predictions by aligning protein sequences from SwissProt (2020-04) to the *C. unifasciata* masked genome with EXONERATE v2.2.0 (Slater and Birney 2005) and by running GeMoMa v1.7.1 (Keilwagen *et al.* 2016, 2018) taking five gastropod species as reference organisms. The selected species were *Pomacea canaliculata* [GCF_003073045.1; (Liu *et al.* 2018)], *Aplysia californica* (GCF_000002075.1), *Elysia chlorotica* [GCA_003991915.1; (Cai *et al.* 2019)]*, Radix auricularia* [GCA_002072015.1; (Schell *et al.* 2017)], and *Chrysomallon squamiferum* [GCA_012295275.1; (Sun *et al.* 2020)]. First, from the mapped RNA-seq reads, introns were extracted and filtered by the GeMoMa modules ERE and DenoiseIntrons. Then, we ran independently the module GeMoMa pipeline for each reference species using mmseqs2 v5877873 (Steinegger and Söding 2017, 2018) and including the mapping of species own RNA-seq data. The five gene annotations were then combined into a final annotation file by using the GeMoMa modules GAF and AnnotationFinalizer. Finally, we aligned *C. unifasciata* transcripts against the masked genome using PASA v2.4.1 (Campbell *et al.* 2006) as implemented in autoAug.pl from Augustus v3.3.3 (Stanke *et al.* 2004).

Gene prediction data from each method were combined using EVidenceMolder v1.1.1 (Haas *et al.* 2008) to obtain a consensus gene set for the *C. unifasciata* genome. Gene models from GeMoMa and SNAP were converted to EVM compatible gff3 files using EVM’s included scripts and combined with CDS identified by TransDecoder into a gene predictions file. After that, EVM was run including gene model predictions, protein and transcript alignments and repeat regions to produce a reliable consensus gene set (Supplementary Table S2).

Predicted genes were functionally annotated by BLAST search against the Swiss-Prot database with an *e*-value cutoff of 10^-6^. InterProScan v5.39.77 (Quevillon *et al.* 2005) was used to predict motifs and domains, as well as Gene ontology (GO) terms.

### Data availability

All raw data generated for this study (Illumina, PacBio, MinION, and RNA-seq reads) are available at the European Nucleotide Archive database (ENA) under the Project number: PRJEB41346. The final genome assembly and annotation can be found under the accession number GCA_905116865.

The execution of this work involved using many software tools which are listed in Table 1. Supplementary File S1 contains BUSCO results. Supplementary File S2 contains *de novo* library of repeats generated by RepeatModeler. Supplementary File S3 contains *C. unifasciata* repeats. Supplementary File S4 contains sequences of protein-coding genes predicted in the *C. unifasciata* genome. Supplementary File S5 contains proteins predicted in the *C. unifasciata genome*. Supplementary File S6 contains genome annotation. Supplementary Table S1 contains a comparison between draft genomes assemblies for *C. unifasciata* obtained by different tools. Supplementary Table S2 contains weights values of evidences for EVM analysis. Supplementary Table S3 contains RepeatMasker results. Supplementary Material is available at figshare: https://doi.org/10.25387/g3.14237828.

**Table 1:** Software employed in this work, their package version and source availability. All url were last accessed on 07-06-2021.

Name	Version	Url
Flye	2.6	https://github.com/fenderglass/Flye
wtdbg2	2.5	https://github.com/ruanjue/wtdbg2
Canu	1.9	https://github.com/marbl/canu
Racon	1.4.3	https://github.com/isovic/racon
Pilon	1.23	https://github.com/broadinstitute/pilon
Quast	5.0.2	https://github.com/ablab/quast
BUSCO	3.0.2	https://busco.ezlab.org/
BlobTools	1.1.1	https://github.com/DRL/blobtools
LINKS	1.8.7	https://github.com/bcgsc/LINKS
Rascaf	1.0.2	https://github.com/mourisl/Rascaf
Long-Read Gapcloser	1.0	https://github.com/CAFS-bioinformatics/LR_Gapcloser
FastQC	0.11.9	https://www.bioinformatics.babraham.ac.uk/projects/fastqc/
Trimmomatic	0.39	http://www.usadellab.org/cms/?page=trimmomatic
MultiQC	1.9	https://multiqc.info/
GenomeScope	1.0	http://qb.cshl.edu/genomescope/
Trinity	2.9.1	https://github.com/trinityrnaseq/trinityrnaseq/wiki
GeMoMa	1.6.4	http://www.jstacs.de/index.php/GeMoMa
MMseqs2	5877873	https://github.com/soedinglab/MMseqs2
Augustus	3.3.3	https://github.com/Gaius-Augustus/Augustus
TransDecoder	5.5.0	https://github.com/TransDecoder
SNAP	2006-07-28	—
EXONERATE	2.2.0	https://www.ebi.ac.uk/about/vertebrate-genomics/software/ exonerate-manual
PASA	2.4.1	https://github.com/PASApipeline/PASApipeline
EVidenceMolder	1.1.1	https://evidencemodeler.github.io
guppy	4.0.11	https://github.com/nanoporetech/pyguppyclient
Nanoplot	1.28.1	https://github.com/wdecoster/NanoPlot
Nanofilt	2.6.0	https://github.com/wdecoster/nanofilt
backmap.pl	0.3	https://github.com/schellt/backmap
SAMtools	1.10	https://github.com/samtools/samtools
BWA	0.7.17	https://github.com/lh3/bwa
minimap2	2.17	https://github.com/lh3/minimap2
Qualimap	2.2.1	http://qualimap.conesalab.org/
bedtools	2.28.0	https://bedtools.readthedocs.io/en/latest/
Rscript	3.6.3	https://www.r-project.org/
RepeatModeler	2.0	http://www.repeatmasker.org/RepeatModeler/
RepeatMasker	4.1.0	http://www.repeatmasker.org/
HISAT2	2.1.0	http://daehwankimlab.github.io/hisat2/

## Results and discussion

### Genome assembly

**Figure 2:**
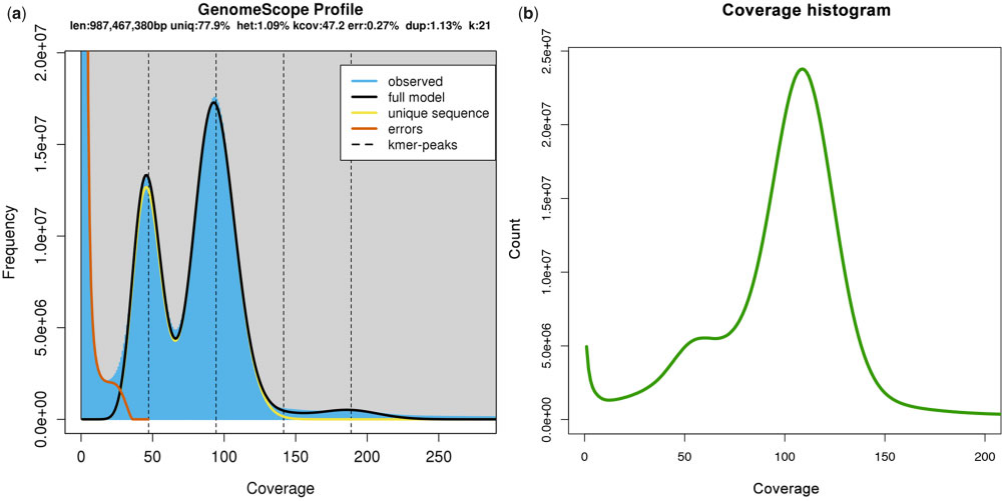
(A) GenomeScope k-mer profile plot for *C. unifasciata* genome. (B) Coverage histogram for the final assembly based on the Illumina reads.

**Figure 3:**
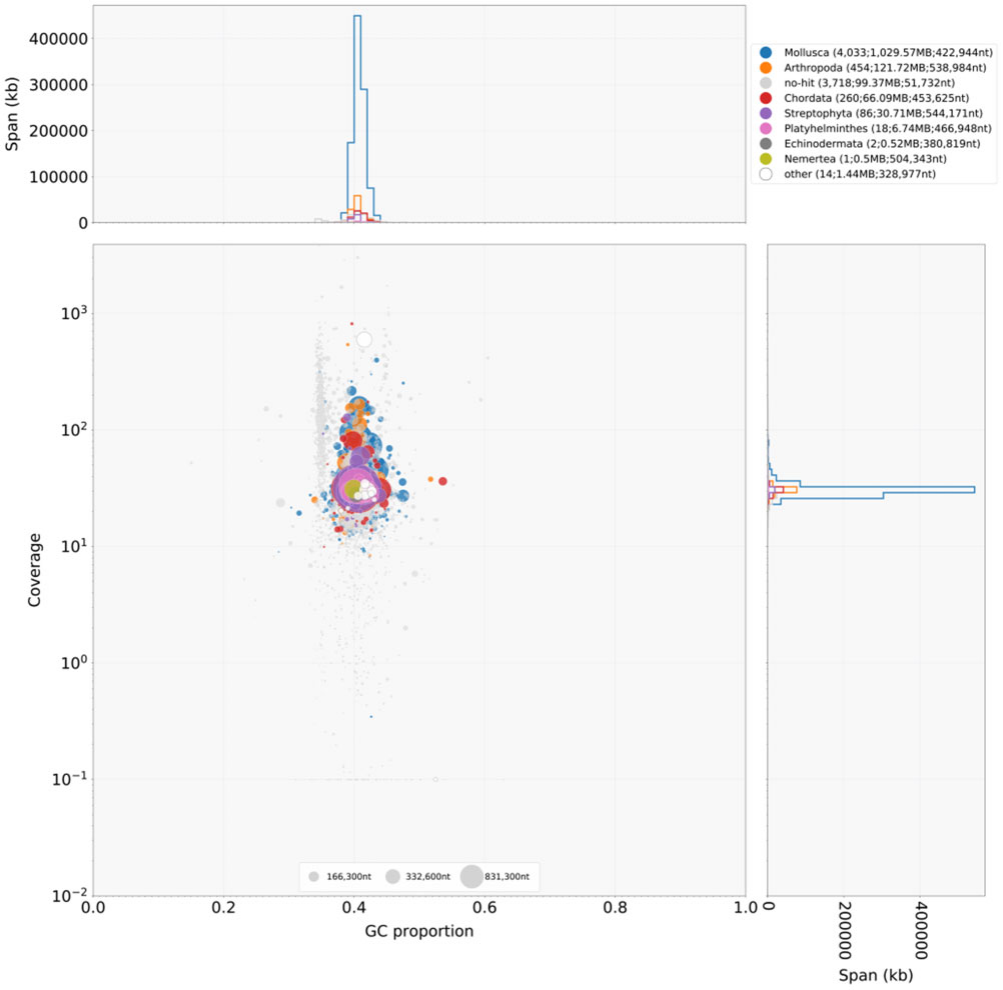
Blob plot showing read coverage, GC content and size of each scaffold. Size of the blobs correspond to size of the scaffold and color corresponds to taxonomic assignment based on a blast search against the nt database.

The calculated DNA content through flow cytometry experiments was 1.54 Gb. The genome size estimation by back-mapped Illumina read coverage resulted in 1.42 Gb. The estimated heterozygosity by GenomeScope of the specimen employed for genome assembly, based on 21-mers, was around 1.09% (Figure 2A), being in the range of other land snail genomes (Guo *et al.* 2019; Saenko *et al.* 2021). We generated sequence data for a total coverage of approximately 120.6X (185.73 Gb), 25.6X (39.43 Gb, N50: 9.9 kb), and 2.1X (3.24 Gb, N50: 16.2 kb) of Illumina, PacBio, and ONT reads respectively. Although we employed HMW-DNA for the ONT sequencing, pores collapsed and started dying very quick, generating less amount of data than expected. After scaffolding with long reads (PacBio and ONT) and RNA data, we produced a draft genome assembly of 1.29 Gb with 8586 scaffolds and a scaffold N50 of 246 kb (Table 2). Contiguity value was also good, with a scaffold NG50 of 341 kb, similar to *C. nemoralis* genome assembly. Completeness evaluation by BUSCO against the metazoan_odb9 data set showed high values, recovering more than the 92% as complete and less than the 6% as missing genes for both, assembly and annotation, analyses (Table 2, Supplementary File S1). These results were in the range of other recent high quality gastropod genome assemblies (Schell *et al.* 2017; Liu *et al.* 2018; Guo *et al.* 2019; Sun *et al.* 2020), being slightly better than closest relative assembly of *C. nemoralis* (Saenko *et al.* 2021). For genome quality evaluation, we compared the *C. unifasciata* draft genome generated with other mollusc genomes publicly available. This comparison showed high quality in terms of contig number and scaffold N50 among land snail genomes. The mapping of the Illumina reads against the final genome assembly showed a mapping rate of 98.56%, as well as a removal of most redundant regions (Figure 2B). Finally, BlobTools analysis shows a small fraction of the assembly assigned to phyla other than Mollusca (*e.g.*, Arthropoda, Chordata among others). This is probably due to incompleteness of the nt database—where the taxonomic assignment is based on—and resulting random hits to more or less conserved sequences throughout the different phyla. Therefore, focusing on GC and coverage distribution, no contamination could be identified (Figure 3), indicating the reliability of the data.

**Table 2:** Genome assembly and annotation statistics for *C. unifasciata* and comparison with other land snails genomes

Statistic	*C. unifasciata*	*C. nemoralis*	*Achatina fulica*
**Total sequence length**	1,286,461,591	3,490,924,950	1,850,322,141
**No. of contigs**	11,756	28,698	8,122
**Contig N50**	246,413	330,079	721,038
**Contig L50**	1,602	3,071	697
**Contig NG50**	205,769	337,823	584,695
**Contig LG50**	2,034	2,964	903
**No. of scaffolds**	8,586	28,537	921
**scaffolds > 10000 bp**	7,180	26,580	189
**Scaffold N50**	246,413	333,110	59,589,303
**Scaffold L50**	940	3,035	13
**Scaffold NG50**	341,667	341,704	58,752,149
**Scaffold LG50**	1,188	2,930	15
**GC content (%)**	40.69	41.25	38.77
**BUSCO**			
**Genome**			
** complete**	92.4% (S : 85.3%; D: 7.1%)	89.0% (S : 73.9%; D: 15.1%)	91.5% (S : 84.6%; D: 6.9%)
** fragmented**	1.6%	3.4%	2.5%
** missing**	6.0%	7.6%	6.0%
**Annotation**			
** complete**	94.5%(S : 86.0%; D : 8.5%)	71.7%(S : 59.5%; D : 12.2%)	95.6%(S : 86.8%; D : 8.8%)
** fragmented**	2.6%	10.4%	1.9%
** missing**	2.9%	17.9%	2.5%
**Transcriptome**		—	—
** complete**	94.7% (S : 52.6%; D: 42.1%)		
** fragmented**	3.8%		
** missing**	1.5%		

### Genome annotation

We estimated the total repeat content of the *C. unifasciata* genome assembly around 61.10% (Table 3, Supplementary Table S3, Supplementary Files S2 and S3), values slightly smaller than other land snails genomes (Guo *et al.* 2019; Saenko *et al.* 2021). Approximately one third of the assembled genome (33.96%) was identified as Transposable elements (TEs) such as long interspersed nuclear elements (LINEs; 25.03%), short interspersed nuclear elements (SINEs; 4.23%), long tandem repeats (LTR; 0.60%), and DNA transposons (4.10%).

**Table 3:** Repeat statistics

Assembly	LINE	SINE	LTR	DNA	Unclassified	SmRNA	Others	Total (%)
***C. unifasciata***	1,253,318	427,509	11,975	298,828	1,334,718	413,197	708,740	61.1
***C. nemoralis***	2,820,864	342,120	209,476	443,363	4,400,828	444,489	1,267,814	76.4

*De novo* and homology based repeat annotations as reported by RepeatMasker and RepeatModeler for *C. unifasciata* and comparison with *C. nemoralis*. Families of repeats included here are long interspersed nuclear elements (LINEs), short interspersed nuclear elements (SINEs), long tandem repeats (LTR), DNA transposons (DNA), unclassified (unknown) repeat families, small RNA repeats (SmRNA), and others (consisting of small, but classified repeat groups). The last column represents the total percentage of base pairs annotated as repeats.

We predicted 22,464 protein coding genes in the *C. unifasciata* genome (Table 4, Supplementary Files S4 and S5) by using a homology-based gene prediction and EVM (Supplementary File S6). Among the identified proteins, 13,221 (62.27%) could be annotated with at least one GO term. Finally, 21,231 proteins (94.51%) were assigned to at least one of the databases used within InterProScan (Table 4). BUSCO and functional annotations results indicated high quality. Total protein-coding genes was in the range of other gastropods annotations (Schell *et al.* 2017; Liu *et al.* 2018; Guo *et al.* 2019), however this number represented only the half of its closest relative *C. nemoralis* (Saenko *et al.* 2021). Nevertheless, *C. nemoralis* genome annotation showed less completeness, with significant numbers of fragmented and missing BUSCO’s. These results could indicate that annotated genes are too fragmented and therefore, the real gene number of *C. nemoralis* annotation might be similar to other gastropods such as *C. unifasciata*.

**Table 4:** Annotation statistics of the predicted protein-coding genes for *C. unifasciata* genome

		*C*. *unifasciata*
**Number**		
	**Gene**	22,464
	**mRNA**	22,464
	**Exon**	147,783
	**CDS**	147,783
**Mean**		
	**mRNAs/gene**	1
	**CDSs/mRNA**	6.58
**Median length**		
	**Gene**	11,931
	**mRNA**	11,931
	**Exon**	129
	**Intron**	2,025
	**CDS**	129
**Total space (Mb)**		
	**Gene**	379,573,459
	**CDS**	26,582,739
**Single**		
	**CDS mRNA**	3,562
**InterproScan**		21,231 (94.51%)
**GO**		13,221 (62.27%)
**Reactome**		5,069 (22.56%)
**SwissProt**		16,809 (74.83%)

## Conclusions

Here, we present a high quality draft assembled and annotated genome of the land snail *C. unifasciata*. The obtained genome is comparable with other land snail and Gastropoda genomes publicly available. The new genome resource will be reference for further population genetics, evolutionary and genomic studies of this highly world-wide diverse group.
